# Radiomics Study for Discriminating Second Primary Lung Cancers From Pulmonary Metastases in Pulmonary Solid Lesions

**DOI:** 10.3389/fonc.2021.801213

**Published:** 2022-01-03

**Authors:** Feiyang Zhong, Zhenxing Liu, Wenting An, Binchen Wang, Hanfei Zhang, Yumin Liu, Meiyan Liao

**Affiliations:** ^1^ Department of Radiology, Zhongnan Hospital of Wuhan University, Wuhan, China; ^2^ Department of Neurology, Zhongnan Hospital of Wuhan University, Wuhan, China

**Keywords:** second primary lung cancers, pulmonary metastases, clinical-radiographic factor, radiomics, lung cancer

## Abstract

**Background:**

The objective of this study was to assess the value of quantitative radiomics features in discriminating second primary lung cancers (SPLCs) from pulmonary metastases (PMs).

**Methods:**

This retrospective study enrolled 252 malignant pulmonary nodules with histopathologically confirmed SPLCs or PMs and randomly assigned them to a training or validation cohort. Clinical data were collected from the electronic medical records system. The imaging and radiomics features of each nodule were extracted from CT images.

**Results:**

A rad-score was generated from the training cohort using the least absolute shrinkage and selection operator regression. A clinical and radiographic model was constructed using the clinical and imaging features selected by univariate and multivariate regression. A nomogram composed of clinical-radiographic factors and a rad-score were developed to validate the discriminative ability. The rad-scores differed significantly between the SPLC and PM groups. Sixteen radiomics features and four clinical-radiographic features were selected to build the final model to differentiate between SPLCs and PMs. The comprehensive clinical radiographic–radiomics model demonstrated good discriminative capacity with an area under the curve of the receiver operating characteristic curve of 0.9421 and 0.9041 in the respective training and validation cohorts. The decision curve analysis demonstrated that the comprehensive model showed a higher clinical value than the model without the rad-score.

**Conclusion:**

The proposed model based on clinical data, imaging features, and radiomics features could accurately discriminate SPLCs from PMs. The model thus has the potential to support clinicians in improving decision-making in a noninvasive manner.

## Introduction

Over the last few decades, owing to advancements in cancer screening and treatment, the life expectancy of cancer survivors continues to improve. It was estimated that approximately 16.9 million Americans were living with cancer as of January 1, 2019, and this number is expected to increase to 20 million by January 1, 2030 (1). Cancer survivors have a higher risk of developing new primary malignant tumors than the general population. The most common newly developed primary malignant tumor is lung cancer (2). Lung cancer remains the leading cause of cancer-related death worldwide (3). Meanwhile, the lungs are the sites most frequently affected by metastasis. Approximately 30% of cancer survivors develop lung metastases (4).

The distinction of second primary lung cancers (SPLCs) from pulmonary metastases (PMs) is of great clinical interest because of the vastly different survival outcomes between them. On account of the close clinical monitoring and regular follow-up of cancer survivors, SPLCs are often diagnosed at an early stage. Compared with primary lung cancer, SPLCs have a fair prognosis after surgical resection of the lesion (5). Both radiotherapy and chemotherapy are regarded as effective methods of treatment (6, 7). Metastasis is the leading cause of mortality among tumor patients (8). The occurrence of metastasis is considered to represent the terminal, incurable stage of a tumor. Early differential diagnosis between these two disorders may help clinicians decide whether aggressive treatment or palliative care is appropriate.

Pathologic assessment remains the gold standard for distinguishing between SPLCs and PMs. Histologically distinct primary tumors are presumed to have diverse origins in a single patient. When tumors are categorized as the same histologic type, immunohistochemistry and genetic testing can assist in confirming the diagnosis (9). However, pathological results cannot be obtained preoperatively. Histopathology specimen acquisition relies on invasive lung biopsy, which may cause several complications, such as pneumothorax, pneumorrhachis, or air embolism. Not all patients are suitable for a needle biopsy or surgical resection. In addition, pathological specimens are typically acquired from one or more separate focal areas and cannot completely characterize the whole tumor.

The value of clinical and imaging characteristics in differential diagnosis between SPLCs and PMs has been reported in our previous study (10) and other articles (11–14). However, there is a lack of radiomics studies concerning the distinctions. Radiomics is an emerging science that extracts a large number of imaging features from radiographic images. It converts images into quantitative parameters and subsequently performs statistical analysis to support decision-making. Previous studies have shown that radiomics can play an important role in diagnosing malignancy, assessing treatment efficacy, and predicting clinical outcomes (15–17). In particular, radiomics has been used to discriminate different pathological types of lung cancer (18, 19). The present study thus aimed to assess whether radiomics features can discriminate SPLCs from PMs and to develop a comprehensive model based on clinical imaging and radiomics to guide clinical decisions.

## Materials and Methods

### Patients

This retrospective, single-center study was approved by the Institutional Review Board of Zhongnan Hospital of Wuhan University and was conducted in accordance with the Declaration of Helsinki. The requirement for informed consent was waived owing to the retrospective nature of this study. The inclusion criteria were as follows: (1) pathological confirmation of malignant pulmonary lesions based on the histopathological evaluation of surgical resection and percutaneous biopsy; (2) thin-section chest CT (section thickness ≤1.5 mm) examination performed within one week before needle biopsy or surgery, and (3) history of malignant tumors. The exclusion criteria were as follows: (1) insufficient image quality for analysis (20), (2) any anti-tumor treatment received before the CT scan, (3) ground-glass opacity (GGO) lesions, (4) uncertainty of whether the lesion was primary or metastatic, and (5) a previous history of multiple primary tumors in separate organs. All pathologically confirmed lesions for included patients were examined unless they had three or more lesions. In this case, the two largest focuses of tumor were selected. Based on the above criteria, 252 lesions (97 SPLCs and 155 PMs) from 245 patients of the given institution from January 2017 to June 2020 were included. Patients included in this study partially were described previously (10). The lesions were randomly assigned to a training cohort (n = 137) or validation cohort (n = 115).

The clinicopathological data, including pathologic assessment, sex, age, history of smoking, family history of malignancy, the recurrence status of the initial tumor, and serum tumor markers [neuron-specific enolase (NSE), carcinoembryonic antigen (CEA), and carbohydrate antigen 125 (CA125)] were obtained by reviewing the electronic medical record system. The upper limit of each tumor marker was the following: NSE, 15.2 ng/mL; CEA, 5 ng/mL; and CA125, 35 U/mL. The above tumor markers were considered positive if their values were higher than the upper limit. Two authors (ZFY and LZX) independently extracted the data.

### CT Scanning

The chest CT images were obtained from the following CT systems: SOMATOM definition scanner (Siemens Healthineers, Forchheim, Germany), and GE discovery 750HD scanner (GE Medical Systems, Milwaukee, WI, USA). The scanning parameters of the above devices were as follows: tube voltage, 120 kV; automatic tube current adjustment technology, 100–350 mAs; matrix size, 512×512; slice interval, 0 mm; standard soft-tissue algorithm reconstruction; reconstructed section thickness, 1 mm.

### Evaluation of Subjective Radiographic Characteristics

The subjective radiographic characteristics were independently analyzed by two thoracic radiologists (FZ, with three years of chest radiological experience, and HZ, with seven years of chest radiological experience), who were blinded to the final pathological results. The CT images were reviewed in the lung window setting (width, 1500 HU; level, −700 HU) and mediastinal window setting (width, 300 HU; level, 40 HU) windows. Discrepancies in the evaluations were resolved through consultation. The imaging characteristics of each pulmonary malignant lesion included (1) lesion size (maximum diameter), (2) distribution of the lesions (single or multiple), (3) central or peripheral type, (4) density (homogeneous or heterogeneous), (5) air bronchogram (absent, present), (6) bubble lucency (absent, present), (7) calcification (absent, present), (8) vessel convergence sign (absent, present), (9) margin (clear, unclear), (10) contour (round, irregular), (11) lobulation (absent, present), (12) spiculation (absent, present), (13) pleural effusion (absent, present), and (14) enlarged mediastinal lymph node (absent, present).

### Region of Interest (ROI) Segmentation and Radiomics Feature Extraction

The pulmonary lesions were semi-automatically segmented using ITK-SNAP (version 3.8.0, http://www.itk-snap.org). The original Digital Imaging and Communications in Medicine files were imported into the in-house software (Analysis Kit, version 3.1.5.R, GE Healthcare) for pre-processing, and the lesions were segmented in standard images, slice by slice, under the lung window setting (width, 1500 HU; level, −700 HU). The lesions were delineated to avoid large vessels, bronchi, and chest walls, if possible.

Radiomics feature extraction was applied to the chest CT images using AK software. Finally, from one segmented ROI, a total of 402 imaging texture features were extracted: 42 histogram features, 144 Gy-level co-occurrence matrix features, 11 Gy-level size zone matrix features, 180 Gy-level run‐length matrix features, and 25 shape- and size-based features. Details of the extraction features are provided in the **Supplementary Material**. Each image was normalized to eliminate the impact of different quantization levels on the texture features.

### Feature Selection and Model Building

Dice Similarity Coefficient was used to describe inter reader segmentation variability, which ranged from 0.74 to 0.98 (**Table S2**) (21). The intraclass correlation coefficient (ICC) was used to evaluate inter-observer agreement of quantitative radiomics parameters. In 20 randomly sampled cases, two chest radiologists (FZ and HZ) independently drew the ROI and extracted the radiomics features. Radiomics features with an ICC higher than 0.75 were regarded as consistent (21) and were included for further analysis. A radiologist (FZ) sketched ROIs in the remaining cases.

In the training set, a minimum redundancy–maximum relevance (mRMR) algorithm was employed to rank the importance of the selected features. Finally, the 100 highest mRMR-ranked features were input to the least absolute shrinkage and selection operator (LASSO) classifier to select the most predictive features. The rad-score was calculated for each lesion based on the final selected features (22), and a receiver operating characteristic (ROC) curve was constructed to evaluate the discriminatory ability of the rad-scores *via* the area under the curve (AUC) in the training and validation cohorts. The clinical data and subjective radiographic characteristics were evaluated using univariate analysis. Significant factors were included in the multivariate analysis to build a clinical–radiographic model.

The clinical–radiographic and rad-scores were combined to construct an individualized discriminatory nomogram based on a multivariate logistic regression algorithm. Internal validation was performed using a calibration curve, which was verified by the Hosmer–Lemeshow test. The AUCs of the ROC were calculated to evaluate the above models in the respective training and validation sets. Decision curve analysis (DCA) was performed to compare the clinical value of the models (23).

### Statistical Analysis

Categorical variables were expressed as frequency rates and compared using the χ² test or Fisher’s exact test. Continuous variables were described as the median (interquartile range [IQR]) and compared using the t-test or Wilcoxon rank-sum test. A two-sided α value of less than 0.05 was considered statistically significant. Statistical analysis was performed using R software (version 4.1.2, http://www.R-project.org). The “mRMRe” package was used to conduct the mRMR algorithm, “irr” package for Intra-class correlation coefficient (ICC) algorithm, “tableone” for comparison of clinical baseline data between groups, “tidyverse” for data collation and exploration, “glmnet” for LASSO regression, “rms” for Nomogram, “rmda” for decision curve analysis(DCA), “pROC” for receiver operating characteristic curve analysis(ROC), “ResourceSelection” for goodness of fit test, and “ggpubr” for data result visualization, respectively.

## Results

### Patient Information

A total of 245 patients (134 men [54.7%] and 111 women [45.3%]) who met the inclusion criteria were included in this retrospective study. The median patient age was 62 years (IQR, 55–67 years; range, 31–93 years). The baseline characteristics of the patients are presented in **Table 1**. Of the 245 patients, 252 solid pulmonary lesions were pathologically diagnosed as malignant foci, including 97 primary lesions and 155 metastatic lesions. There are 21 synchronous SPLCs and 76 metachronous ones. 55SPLCs and 82 PMs were included in the training set, while 42 SPLCs and 73 PMs were included in the validation set. Univariate analysis revealed no difference between the clinical data and subjective radiographic characteristics in the training and validation sets.

**Table 1:** The clinical and radiographic factors of patients in SPLC^†^ and PM^§^ Groups.

Variables	SPLC Group (N=97)	PM Group (N=155)	P value
	No. of patient (%)	No. of patient (%)	
Sex			0.029
Male	62 (63.9)	76 (49.0)	
Female	35 (36.1)	79 (51.0)	
Age (years)			<0.001
(Median [IQR])	64.00 [59.00, 68.00]	59.00 [52.00, 65.00]	
History of smoking			0.003
Yes	54 (55.7)	116 (74.8)	
No	43 (44.3)	39 (25.2)	
Family history of malignancy (%)			0.43
Yes	88 (90.7)	146 (94.2)	
No	9 (9.3)	9 (5.8)	
Recurrence status of the initial tumor			0.085
Yes	97 (100.0)	149 (96.1)	
No	0 (0.0)	6 (3.9)	
Maximal lesion size (mm)			<0.001
(Median [IQR])	30.00 [20.00, 49.00]	19.00 [13.00, 28.50]	
NSE			0.003
Normal	74 (76.3)	141 (91.0)	
Abnormal	23 (23.7)	14 (9.0)	
CEA			0.002
Normal	59 (60.8)	123 (79.4)	
Abnormal	38 (39.2)	32 (20.6)	
CA125			0.034
Normal	69 (71.1)	129 (83.2)	
Abnormal	28 (28.9)	26 (16.8)	
The distribution of lesions			<0.001
Single	80 (82.5)	72 (46.5)	
Multiple	17 (17.5)	83 (53.5)	
Central or peripheral type			<0.001
Peripheral	75 (77.3)	149 (96.1)	
Central	22 (22.7)	6 (3.9)	
Density			0.546
Homogeneous	79 (81.4)	120 (77.4)	
Heterogeneous	18 (18.6)	35 (22.6)	
Air bronchogram			<0.001
Absent	47 (48.5)	134 (86.5)	
Present	50 (51.5)	21 (13.5)	
Bubble lucency			0.283
Absent	81 (83.5)	138 (89.0)	
Present	16 (16.5)	17 (11.0)	
Calcification			0.001
Absent	79 (81.4)	147 (94.8)	
Present	18 (18.6)	8 (5.2)	
Vessel convergence sign			<0.001
Absent	62 (63.9)	137 (88.4)	
Present	35 (36.1)	18 (11.6)	
Margin			0.003
Clear	79 (81.4)	146 (94.2)	
Unclear	18 (18.6)	9 (5.8)	
Contour			<0.001
Round	22 (22.7)	118 (76.1)	
Irregular	75 (77.3)	37 (23.9)	
Lobulation			<0.001
Absent	6 (6.2)	40 (25.8)	
Present	91 (93.8)	115 (74.2)	
Spiculation			<0.001
Absent	30 (30.9)	130 (83.9)	
Present	67 (69.1)	25 (16.1)	
Pleural effusion			<0.001
Absent	25 (25.8)	83 (53.5)	
Present	72 (74.2)	72 (46.5)	
Enlarged mediastinal lymph node			0.158
Absent	70 (72.2)	125 (80.6)	
Present	27 (27.8)	30 (19.4)	

^†^SPLC indicates second primary lung cancer; ^§^PM, pulmonary metastasis.

### Comparison of Clinical and Radiographic Features Between SPLC and PM Groups

The univariate logistic regression analysis of the clinical data showed a statistically significant difference in sex, history of smoking, and CEA level between the SPLC and PM groups in the training set (p <0.05). Other clinical data, such as age, recurrence status of the initial tumor, family history of malignancy, NSE level, and CA125 level, were not statistically significant.

Among the visual imaging findings, the maximum diameter of lesions, the distribution of lesions, central or peripheral type of lesions, air bronchogram, calcification, vessel convergence sign, contour, lobulation, spiculation, and pleural effusion presented significant differences in the univariate analysis (p <0.05). Other radiographic features, such as density, bubble lucency, margin, and enlarged mediastinal lymph nodes, were not statistically significant in the two groups. All clinical–radiographic variables that achieved statistical significance were enrolled in the multivariate logistic regression analysis.

In the multivariate analysis, the distribution of lesions (odds ratio [OR], 6.52; 95% confidence interval [CI], 1.92–26.84; p-value = 0.005), central or peripheral type (OR, 0.05; 95% CI, 0–0.66; p-value = 0.031), contour (OR, 0.23; 95% CI, 0.06–0.76; p-value = 0.018), and spiculation (OR, 0.12; 95% CI, 0.03–0.42; p-value <0.001) were identified as independent variables and included in the clinical–radiographic model.

### Feature Selection and Radiomics Model Building

**Figure 1:**
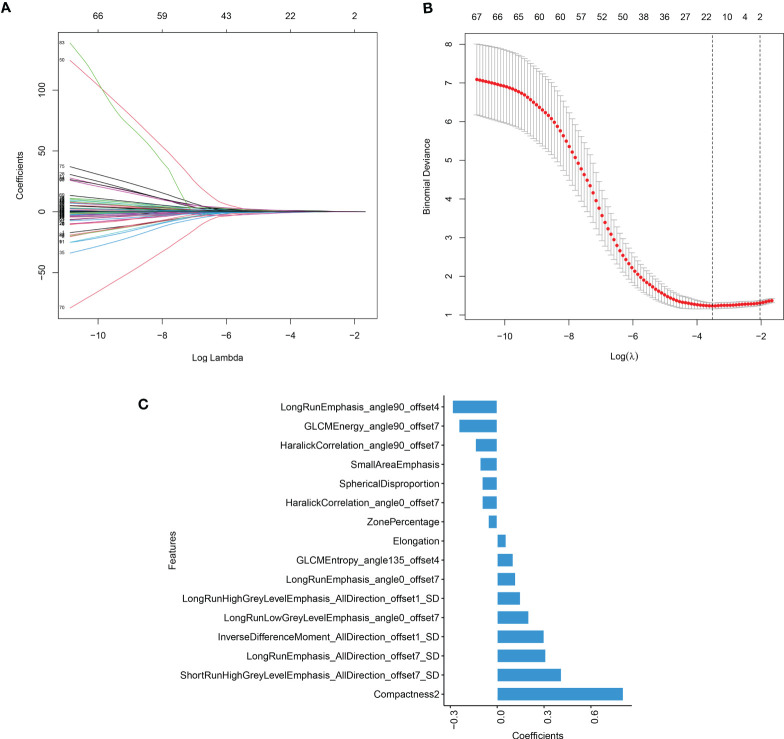
The result of LASSO model **(A)** LASSO coefficient profiles of the candidate predictors. **(B)** The features with nonzero coefficients are shown in the model. **(C)** The y-axis indicates the selected radiomics features, and the x-axis represents the coefficient of radiomics.

A total of 233 radiomics features with an ICC higher than 0.75 were enrolled for the next feature extraction step. Then, the mRMR program was used to select the 100 highest-ranked features in the training set. Finally, LASSO logistic regression was used to reduce the 100 features to 16 features with nonzero coefficients, as shown in **Figure 1**. The rad-score of each lesion was calculated using the following formula:


$$\begin{array}{l} \text{Rad}-\text{Score}\;=\;0.585917936\;+\;0.300121336\;\times \;\text{InverseDifferenceMoment}\_\text{AllDirection}\_\text{offset}1\_\text{SD}\\ +\;0.139511141\;\times \;\text{HaralickCorrelation}\_\text{angle}90\_\text{offset}7\;+\;0.117363006\\ \times \;\text{LongRunEmphasis}\_\text{angle}0\_\text{offset}7\;-\;0.286189566\;\times \;\text{LongRunEmphasis}\_\text{angle}0\_\text{offset}4\\ +\;0.807308895\;\times \;\text{Compactness}2\;-\;0.095952964\;\times \;\text{HaralickCorrelation}\_\text{angle}0\_\text{offset}7\;-\;0.09620897\\ \times \;\text{SphericalDisproportion}\;+\;0.410874754\\ \times \;\text{ShortRunHighGreyLevelEmphasis}\_\text{AllDirection}\_\text{offset}7\_\text{SD}\;-\;0.109558249\;\times \;\text{SmallAreaEmphasis}\\ -\;0.05680965\;\times \;\text{ZonePercentage}\;+\;0.102430665\;\times \;\text{GLCMEntropy}\_\text{angle}135\_\text{offset}4\;+\;0.148896463\\ \times \;\text{LongRunHighGreyLevelEmphasis}\_\text{AllDirection}\_\text{offset}1\_\text{SD}\;+\;0.057282871\;\times \;\text{Elongation}\\ +\;0.202108196\times \text{LongRunLowGreyLevelEmphasis}\_\text{angle}0\_\text{offset}7\;-\;0.244924038\\ \times \;\text{GLCMEnergy}\_\text{angle}90\_\text{offset}7\;+\;0.310948511\;\times \;\text{LongRunEmphasis}\_\text{AllDirection}\_\text{offset}7\_\text{SD} \end{array}$$

**Figure 2:**
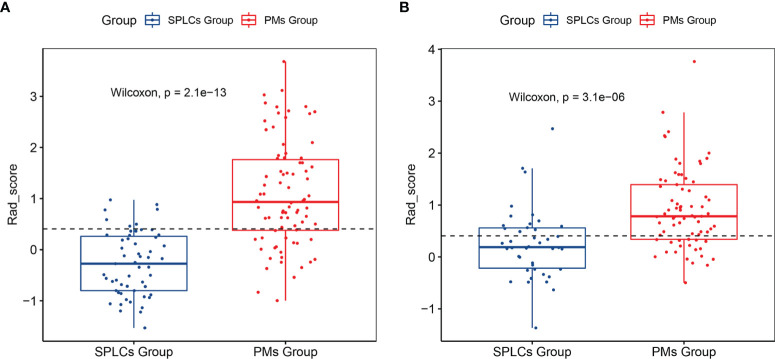
The Rad-score of each lesion in the training set **(A)** and validation set **(B)**.

The rad-scores were significantly different between the SPLC and the PM groups in both the training and validation sets (p <0.05); PMs had higher rad-scores than SPLCs. The rad-scores for both the training and validation sets are shown in **Figure 2**.

### Nomogram Model Construction and Validation

**Figure 3:**
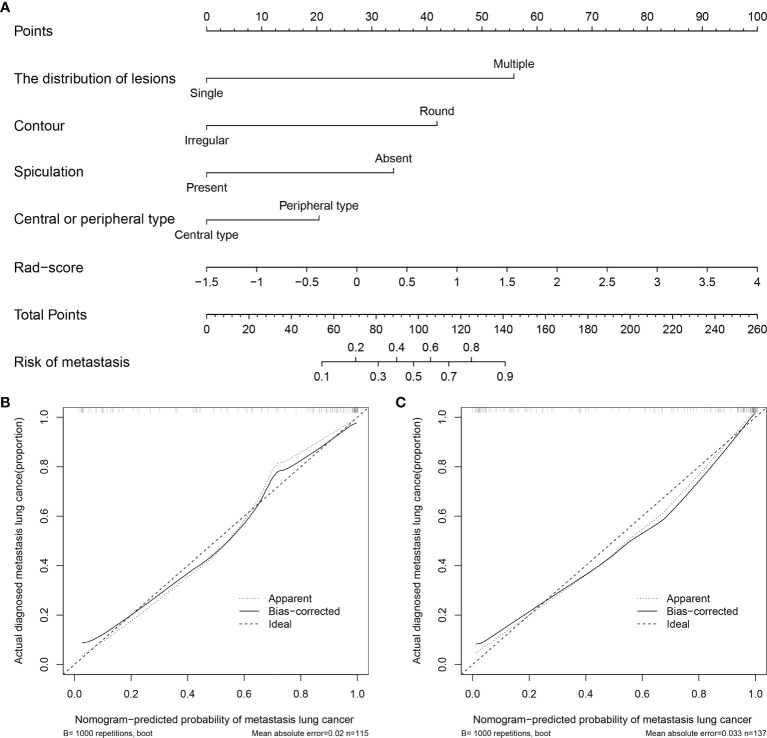
**(A)** Nomogram for predicting SPLCs and PMs. For each patient, draw a vertical line between the variable value and the corresponding point line, and then assign a score for each variable based on the clinical and imaging characteristics to obtain a total score. The risk of metastasis can be predicted according to the total score. **(B)** Calibration curve for the nomogram in training cohort. **(C)** Calibration curve for the nomogram in validation cohort.

**Figure 4:**
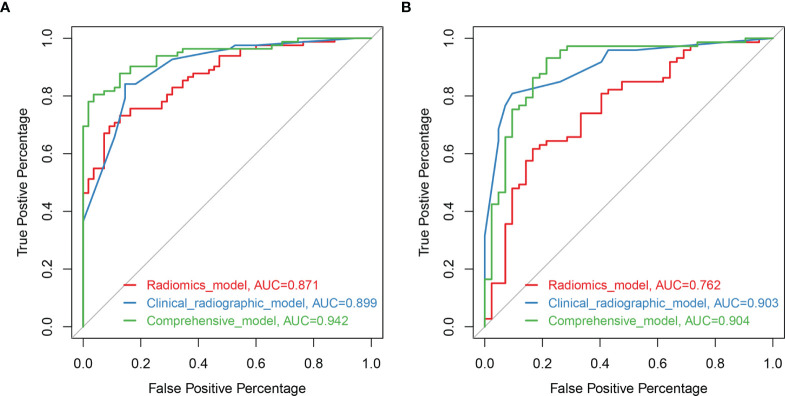
Receiver operating characteristic (ROC) curves of the models based on clinical-radiographic factors (blue), radiomics features alone (red), and comprehensive clinical-radiography-radiomics features (green) in the training set **(A)** and validation set **(B)**.

**Figure 5:**
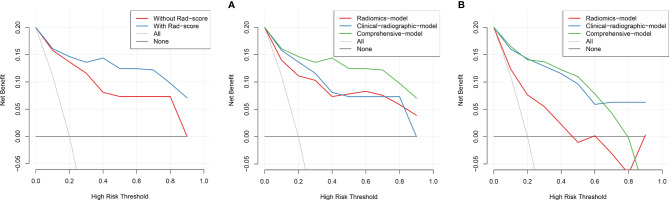
The DCA showed that in most circumstances, using the comprehensive model to distinguish between SPLCs and PMs would be more clinically beneficial than other models in training cohort **(A)** and validation cohort **(B)**.

Based on the training set, the radiomics scores, distribution of lesions, central or peripheral type, contour, and spiculation were incorporated into the comprehensive nomogram model construction (**Figure 3A**). **Figure 3** shows the calibration curves of the nomogram model in training cohort (B) and validation cohort (C). The Hosmer–Lemeshow test finding was not significant (P = 0.4612); it showed good calibration in the training set. **Figure 4** shows the discriminative abilities of each model. The radiomics model had good discriminative performance, with AUCs of 0.8707 (95% CI, 0.8138–0.9277) in the training set and 0.7622 (95% CI, 0.6702–0.8543) in the validation set. The clinical–radiographic model had AUCs of 0.8989 (95% CI, 0.8475–0.9503) and 0.9035 (95% CI, 0.8489–0.9581) in the training and validation cohorts, respectively. The comprehensive model achieved a slightly higher AUC in the training (0.9421; 95% CI, 0.9056–0.9786) and validation sets (0.9041; 95% CI, 0.8417–0.9665). **Figure 5** presents the DCA of the nomogram. The DCA showed that in most circumstances, using the comprehensive model to distinguish between SPLCs and PMs would be more clinically beneficial than using other models in training cohort (A) and validation cohort (B).

## Discussion

In this study, the ability of CT-based radiomics to discriminate SPLCs from PMs was investigated. An individual nomogram model integrated with clinical data, radiographic characteristics, and radiomics features was constructed. It achieved an excellent discriminative capability and has the potential to support clinicians in improving decision-making in a noninvasive manner.

In recent years, radiomics studies have attracted increasing attention because they can reflect quantitative intratumoral pathophysiological information in a noninvasive manner (24). Radiomics features represented tumor heterogeneity and were extracted from the entire ROI; they were not just limited to the biopsy site (17). Previous studies demonstrated that radiomics plays a role in differentiating between primary and metastatic tumors (25–29). In particular, CT radiomics features combined with positron emission tomography (PET) features can accurately distinguish between primary and metastatic lung cancers (26, 27). However, neither of these previous studies focused on the patient’s history of neoplastic disease, leading to insufficient clinical value. To the best of the present authors’ knowledge, this is the first study to apply radiomics studies to SPLCs and to build a radiomics model for distinguishing SPLCs from PMs. In this study, the rad-scores in the SPLC group were significantly lower than those in the PM group (−0.005 [IQR −0.516–0.365] *vs*. 0.830 [IQR 0.357–1.53]), thereby showing strong diagnostic efficacy.

Several studies have demonstrated that combining radiographic and radiomics provides a higher prognostic performance than radiomics alone in lung lesions (20, 30, 31). Despite some observer bias, the subjective radiographic characteristic is the most frequently used method for describing pulmonary lesions. Pulmonary GGO lesions have shown that tumor cells grow along the alveolar wall and are known to be a key sign of primary lung adenocarcinoma (11, 14, 32). This was also observed at the present research institution (10); therefore, only solid lesions were employed in this study. A prior study argue that central-type pulmonary lesion strongly prompts to be the SPLC because endobronchial metastasis is a rare event (13), and the same result was obtained in this study. In the final model, four independent imaging characteristics, including the distribution of lesions, central or peripheral type, contour, and spiculation, were in accordance with the authors’ clinical experience. The final model did not include clinical variables because they were not statistically significant in the multiple logistic regression analysis. In the past, the event-free duration is considered to be an important differentiating factor (13). However, it is difficult to accurately measure the time between primary and secondary tumor. So, this characteristic was not included in this study. Smoking history used to be a risk factor for SPLC (14, 33) but it has not been seen in this research. This may be because traditional Chinese women smoke less. Thus, combining the radiomics model with the radiographic features improved the prognostic performance.

However, there were some limitations to this study. First, it was a retrospective, single-center study with a small sample size. As the number of cases was small, different organs of initial primary cancers were not distinguished, which may have led to bias. Therefore, larger sample sizes from multiple centers are required for further studies. Second, radiomics feature extraction was performed only on plain CT scanning images. Enhanced CT or PET images may contain added valuable information. Third, in this study, a semi-automatic method was adopted to segment ROIs, which could lead to artificial differences. An accurate automatic segmentation method should be considered in future studies (34). Last, the relationship between radiomics signatures and subjective radiographic characteristics was not assessed. This aspect will be explored in future work.

In conclusion, the model developed using clinical–radiographic factors and CT-based radiomics features shows good performance discriminating between SPLCs and PMs. Therefore, for pulmonary malignancy patients with a history of other malignant tumors, the individual nomogram model may guide therapeutic decisions. With the development of artificial intelligence and machine learning, quantitative radiomics may have promising clinical applications.

## Data Availability Statement

The original contributions presented in the study are included in the article/**Supplementary Material**. Further inquiries can be directed to the corresponding author.

## Ethics Statement

The studies involving human participants were reviewed and approved by Zhongnan Hospital of Wuhan University. Written informed consent for participation was not required for this study in accordance with the national legislation and the institutional requirements.

## Author Contributions

FZ and ZL designed the study, analyzed the data, and wrote the manuscript. WA, BW, and HZ participated in data collection and processing. ML and YL reviewed the manuscript. All authors contributed to the article and approved the submitted version.

## Conflict of Interest

The authors declare that the research was conducted in the absence of any commercial or financial relationships that could be construed as a potential conflict of interest.

## Publisher’s Note

All claims expressed in this article are solely those of the authors and do not necessarily represent those of their affiliated organizations, or those of the publisher, the editors and the reviewers. Any product that may be evaluated in this article, or claim that may be made by its manufacturer, is not guaranteed or endorsed by the publisher.

## References

[1] Miller KD Nogueira L Mariotto AB Rowland JH Yabroff KR Alfano CM. Cancer Treatment and Survivorship Statistics, 2019. CA: Cancer J Clin (2019) 69(5):363–85. doi: 10.3322/caac.21565 31184787

[2] Araujo-Filho JB Halpenny D Mcquade C Puthoff G Chiles C Nishino M. Management of Pulmonary Nodules in Oncologic Patients: Expert Panel Narrative Review. AJR Am J Roentgenol (2021) 216(6):1423–31. doi: 10.2214/AJR.20.24907 33355489

[3] Siegel RL Miller KD Jemal A. Cancer Statistics, 2020. CA: Cancer J Clin (2020) 70(1):7–30. doi: 10.3322/caac.21590 31912902

[4] Hong Y Li Z Zhang Q. A Circulating Tumor Cell Cluster-Based Model for Tumor Metastasis (Hypothesis). Oncol Lett (2016) 12(6):4891–5. doi: 10.3892/ol.2016.5358 PMC5228435 28105198

[5] Ko K-H Huang H-K Chen Y-I Chang H Tsai W-C Huang T-W. Surgical Outcomes of Second Primary Lung Cancer After the Extrapulmonary Malignancy. J Cancer Res Clin Oncol (2020) 146(12):3323–32. doi: 10.1007/s00432-020-03310-x PMC11804703 32632580

[6] Wu Y Han C Chong Y Liu J Gong L Wang Z. Prognostic Study for Survival Outcome Following the Treatment of Second Primary Lung Cancer in Patients With Previously Resected Non-Small Cell Lung Cancer. Thorac Cancer (2020) 11(10):2840–51. doi: 10.1111/1759-7714.13610 PMC7529572 32851789

[7] Wang Z Wu Y Wang L Gong L Han C Xie F. Role of Chemotherapy for Survival in Patients With Second Primary Non-Small Cell Lung Cancer. Thorac Cancer (2021) 12(4):426–43. doi: 10.1111/1759-7714.13762 PMC7882385 33295696

[8] Li Y-L Chen C-H Chen J-Y Lai YS Wang SC Jiang SS. Single-Cell Analysis Reveals Immune Modulation and Metabolic Switch in Tumor-Draining Lymph Nodes. Oncoimmunology (2020) 9(1):1830513. doi: 10.1080/2162402X.2020.1830513 33117603 PMC7575008

[9] Detterbeck FC Franklin WA Nicholson AG Girard N Arenberg DA Travis WD. The IASLC Lung Cancer Staging Project: Background Data and Proposed Criteria to Distinguish Separate Primary Lung Cancers From Metastatic Foci in Patients With Two Lung Tumors in the Forthcoming Eighth Edition of the TNM Classification for Lung Cancer. J Thorac Oncol Off Publ Int Assoc Study Lung Cancer (2016) 11(5):651–65. doi: 10.1016/j.jtho.2016.01.025 26944304

[10] Zhong F Liu Z Wang B An W Zhang H Liao M. A Predictive Model to Differentiate Between Second Primary Lung Cancers and Pulmonary Metastasis. Acad Radiol (2021) S1076-6332(21)00254–3. doi: 10.1016/j.acra.2021.05.015 34175210

[11] Ohtaki Y Shimizu K Nagashima T Nakazawa S Obayashi K Azuma Y. Clinical and Radiological Discrimination of Solitary Pulmonary Lesions in Colorectal Cancer Patients. World J Surg (2018) 42(4):1161–70. doi: 10.1007/s00268-017-4243-9 28983707

[12] Dijkman BG Schuurbiers OCJ Vriens D Looijen-Salamon M Bussink J Timmer-Bonte JN. The Role of (18)F-FDG PET in the Differentiation Between Lung Metastases and Synchronous Second Primary Lung Tumours. Eur J Nucl Med Mol Imaging (2010) 37(11):2037–47. doi: 10.1007/s00259-010-1505-2 PMC2948164 20533031

[13] Ge J Gou H-F Chen Y Cheng K Li LH Dong H. Clinical Characteristics of Patients With Solitary Pulmonary Mass After Radical Treatment for Primary Cancers: Pulmonary Metastasis or Second Primary Lung Cancer? Cancer Invest (2013) 31(6):397–403. doi: 10.3109/07357907.2013.800092 23758185

[14] Nakadate A Nakadate M Sato Y Nakagawa T Yoshida K Suzuki Y. Predictors of Primary Lung Cancer in a Solitary Pulmonary Lesion After a Previous Malignancy. Gen Thorac Cardiovasc Surg (2017) 65(12):698–704. doi: 10.1007/s11748-017-0825-6 28887727

[15] Gillies RJ Kinahan PE Hricak H. Radiomics: Images Are More Than Pictures, They Are Data. Radiology (2016) 278(2):563–77. doi: 10.1148/radiol.2015151169 PMC4734157 26579733

[16] Bi WL Hosny A Schabath MB Giger ML Birkbak NJ Mehrtash A. Artificial Intelligence in Cancer Imaging: Clinical Challenges and Applications. CA: Cancer J Clin (2019) 69(2):127–57. doi: 10.3322/caac.21552 PMC6403009 30720861

[17] Mayerhoefer ME Materka A Langs G Häggström I Szczypinski P Gibbs P. Introduction to Radiomics. J Nucl Med (2020) 61(4):488–95. doi: 10.2967/jnumed.118.222893 PMC9374044 32060219

[18] Chen BT Chen Z Ye N Mambetsariev I Fricke J Daniel E. Differentiating Peripherally-Located Small Cell Lung Cancer From Non-Small Cell Lung Cancer Using a CT Radiomic Approach. Front Oncol (2020) 10:593–3. doi: 10.3389/fonc.2020.00593 PMC7188953 32391274

[19] Liu S Liu S Zhang C Yu H Liu X Hu Y. Exploratory Study of a CT Radiomics Model for the Classification of Small Cell Lung Cancer and Non-Small-Cell Lung Cancer. Front Oncol (2020) 10:1268–8. doi: 10.3389/fonc.2020.01268 PMC7498676 33014770

[20] Kim HY Shim YM Lee KS Han J Yi CA Kim YK. Persistent Pulmonary Nodular Ground-Glass Opacity at Thin-Section CT: Histopathologic Comparisons. Radiology (2007) 245(1):267–75. doi: 10.1148/radiol.2451061682 17885195

[21] Wu S Zheng J Li Y Yu H Shi S Xie W. A Radiomics Nomogram for the Preoperative Prediction of Lymph Node Metastasis in Bladder Cancer. Clin Cancer Res (2017) 23(22):6904–11. doi: 10.1158/1078-0432.CCR-17-1510 28874414

[22] Huang Y Liu Z He L Chen X Pan D Ma Z. Radiomics Signature: A Potential Biomarker for the Prediction of Disease-Free Survival in Early-Stage (I or II) Non-Small Cell Lung Cancer. Radiology (2016) 281(3):947–57. doi: 10.1148/radiol.2016152234 27347764

[23] Vickers AJ Elkin EB. Decision Curve Analysis: A Novel Method for Evaluating Prediction Models. Med Decis Making (2006) 26(6):565–74. doi: 10.1177/0272989X06295361 PMC2577036 17099194

[24] Zhu X Dong D Chen Z Fang M Zhang L Song J. Radiomic Signature as a Diagnostic Factor for Histologic Subtype Classification of Non-Small Cell Lung Cancer. Eur Radiol (2018) 28(7):2772–8. doi: 10.1007/s00330-017-5221-1 29450713

[25] Hu Y Weng Q Xia H Chen T Kong C Chen W. A Radiomic Nomogram Based on Arterial Phase of CT for Differential Diagnosis of Ovarian Cancer. Abdom Radiol (NY) (2021) 46(6):2384–92. doi: 10.1007/s00261-021-03120-w PMC8205899 34086094

[26] Kirienko M Cozzi L Rossi A Voulaz E Antunovic L Fogliata A. Ability of FDG PET and CT Radiomics Features to Differentiate Between Primary and Metastatic Lung Lesions. Eur J Nucl Med Mol Imaging (2018) 45(10):1649–60. doi: 10.1007/s00259-018-3987-2 29623375

[27] Zhou Y Ma X-L Zhang T Wang J Zhang T Tian R. Use of Radiomics Based on F-FDG PET/CT and Machine Learning Methods to Aid Clinical Decision-Making in the Classification of Solitary Pulmonary Lesions: An Innovative Approach. Eur J Nucl Med Mol Imaging (2021) 48(9):2904–13. doi: 10.1007/s00259-021-05220-7 33547553

[28] Lenga L Bernatz S Martin SS Booz C Solbach C Mulert-Ernst R. Iodine Map Radiomics in Breast Cancer: Prediction of Metastatic Status. Cancers (Basel) (2021) 13(10):2431. doi: 10.3390/cancers13102431 34069795 PMC8157278

[29] Mao B Ma J Duan S Xia Y Tao Y Zhang L. Preoperative Classification of Primary and Metastatic Liver Cancer *via* Machine Learning-Based Ultrasound Radiomics. Eur Radiol (2021) 31(7):4576–86. doi: 10.1007/s00330-020-07562-6 33447862

[30] Wen Q Yang Z Dai H Feng A Li Q. Radiomics Study for Predicting the Expression of PD-L1 and Tumor Mutation Burden in Non-Small Cell Lung Cancer Based on CT Images and Clinicopathological Features. Front Oncol (2021) 11:620246–6. doi: 10.3389/fonc.2021.620246 PMC8377473 34422625

[31] Shao X Niu R Shao X Jiang Z Wang Y. Value of (18)F-FDG PET/CT-Based Radiomics Model to Distinguish the Growth Patterns of Early Invasive Lung Adenocarcinoma Manifesting as Ground-Glass Opacity Nodules. EJNMMI Res (2020) 10(1):80–0. doi: 10.1186/s13550-020-00668-4 PMC7359213 32661639

[32] Suh YJ Lee H-J Sung P Yoen H Kim S Han S. A Novel Algorithm to Differentiate Between Multiple Primary Lung Cancers and Intrapulmonary Metastasis in Multiple Lung Cancers With Multiple Pulmonary Sites of Involvement. J Thorac Oncol: Off Publ Int Assoc Study Lung Cancer (2020) 15(2):203–15. doi: 10.1016/j.jtho.2019.09.221 31634666

[33] Boyle JM Tandberg DJ Chino JP D’amico TA Ready NE Kelsey CR. Smoking History Predicts for Increased Risk of Second Primary Lung Cancer: A Comprehensive Analysis. Cancer (2015) 121(4):598–604. doi: 10.1002/cncr.29095 25283893

[34] Huang L Xia W Zhang B Qiu B Gao X. MSFCN-Multiple Supervised Fully Convolutional Networks for the Osteosarcoma Segmentation of CT Images. Comput Methods Programs BioMed (2017) 143:67–74. doi: 10.1016/j.cmpb.2017.02.013 28391820

